# The Buccal Pedicle Sliding Flap Technique for Keratinized Tissue Augmentation During the Second-Stage Surgery: A Report of Two Cases

**DOI:** 10.7759/cureus.46362

**Published:** 2023-10-02

**Authors:** Pratha Akolu, Priya Lele, Vidya Dodwad, Manasi Yewale

**Affiliations:** 1 Department of Periodontology, Bharati Vidyapeeth (Deemed to Be University) Dental College and Hospital, Pune, IND

**Keywords:** periodontal phenotype, palatal tissue, inadequate keratinized tissue, peri-implant tissues, second stage surgery, implant

## Abstract

The need for adequacy of keratinized tissue (KT) around dental implants has been a topic of debate over the past few years. Peri-implant tissues differ from those around natural teeth. Therefore, the requirement for healthy peri-implant tissue is of importance. There is general agreement that a thick zone of KT around implants promotes accurate prosthetic procedures, permits maintenance of oral hygiene, resists recession and enables esthetic blending with surrounding tissues. Soft tissue augmentation around implants, when required, can be performed at various stages of implant therapy. The second stage of surgery involves the uncovery of the implant and placement of the healing abutment of desired collar height to achieve a biologic seal around the implant. It can be performed either by excision or by incision depending upon the clinical situation. This stage is a golden opportunity for the implant surgeon to modify the periodontal phenotype around the implant if need be. Different procedures such as palatal roll flap, rotated pedicle flap, free gingival graft, etc. can be performed to increase the keratinized tissue width (KTW) around implants. This case series demonstrates a novel minimally invasive technique to augment the KT in the maxillary arch during the second stage of surgery.

## Introduction

Over the last two decades, dental implants have become an indispensable part of dental treatment. It has been accepted that implant therapy is a prosthetically driven treatment option which requires adequate bone volume, optimal soft tissue profile and harmonious occlusion for long-lasting successful outcomes. Traditionally, the mainstay of implant planning was hard tissue assessment to achieve successful osseointegration. Recently it has been identified that the peri-implant soft tissue plays a fundamental role that assures the long-term survival of an implant. Therefore soft tissue evaluation (thickness, width of keratinized tissue (KT), vestibular depth, frenal pull, etc.) at the potential implant site is crucial.

The peri-implant tissues differ from the gingiva around the natural tooth mainly in three aspects. The absence of periodontal ligament around an implant leads to loss of proprioception and also compromises the vasculature and direct anchorage to the implant surface. More number of collagen fibres and fibre orientation parallel to the implant surface weakens the quality of attachment and makes it more vulnerable to mechanical insults. The peri-implant tissue thus resembles scar tissue. Due to these differences, the presence of healthy soft tissue around the implant is important.

However, the necessity of keratinized mucosa around dental implants has been debated in literature. Controversy exists with respect to the question of whether or not there is a need to augment the KT around implants in patients with a lack of adequate width or thickness. Lang and Loe in their longitudinal clinical study concluded that 2 mm of keratinized tissue width (KTW), including 1 mm of attached gingiva is adequate to maintain gingival health [1]. Chung et al. [2] reported that the absence of an adequate amount of keratinized mucosa around dental implants, especially in the posterior region, was associated with higher plaque accumulation and gingival inflammation. This was in agreement with a cross-sectional study done by Bouri et al. [3] who concluded that increased width of keratinized mucosa (>2 mm) around implants is associated with lower mean alveolar bone loss and improved indices of soft tissue health.

Esposito et al. [4] in their Cochrane systematic review highlighted that the presence of KT around implants is certainly paramount from a clinical perspective to simplify a patient’s oral hygiene maintenance and mucosal tissue stability.

Soft tissue augmentation to preserve or enhance the peri-implant tissues is consequential for the restorative as well as esthetic outcome. There are four potential stages during which soft tissue augmentation can be approached i.e. before and during implant placement, during second-stage surgery, and finally during the maintenance phase [5]. In two-piece implants with delayed loading protocol, the second stage of surgery involves uncovering the dental implant and connecting the healing abutment to the fixture. It aims to develop a healthy peri-implant seal in addition to exposing the implant to the necessary restorative procedures [6].

The maxilla offers a significant benefit due to the availability of abundant palatal KT, which can be rotated or repositioned for augmenting the zone of KM around implants. This case series aims to demonstrate a novel minimally invasive technique - a buccal pedicle sliding flap, incorporating the palatal tissue, performed during second-stage surgery to increase the amount of KT around maxillary implants.

## Case presentation

Case 1

**Figure 1:**
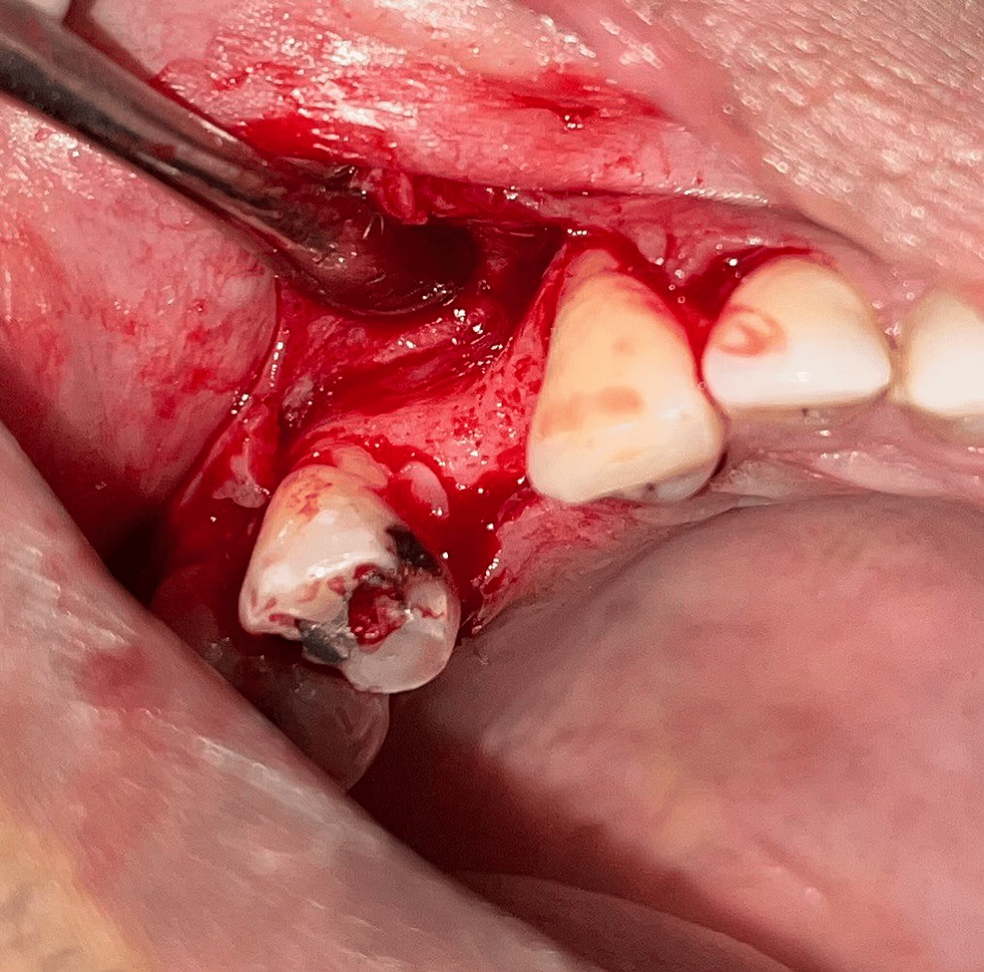
Case 1 - Horizontal ridge defect seen in the edentulous region of 14 after flap reflection

**Figure 2:**
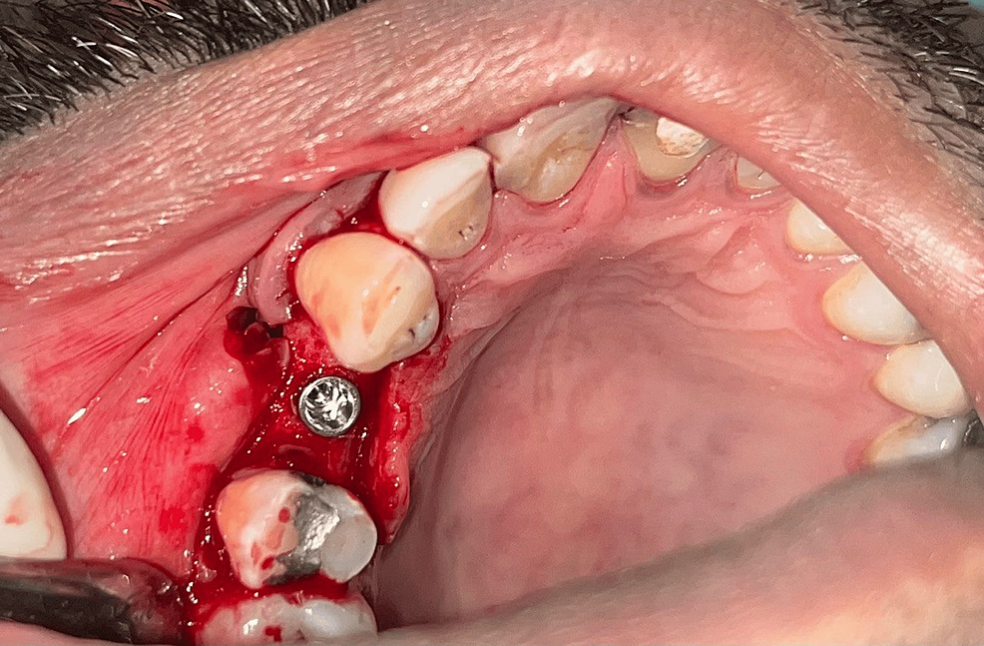
Case 1 - Dental implant placement

**Figure 3:**
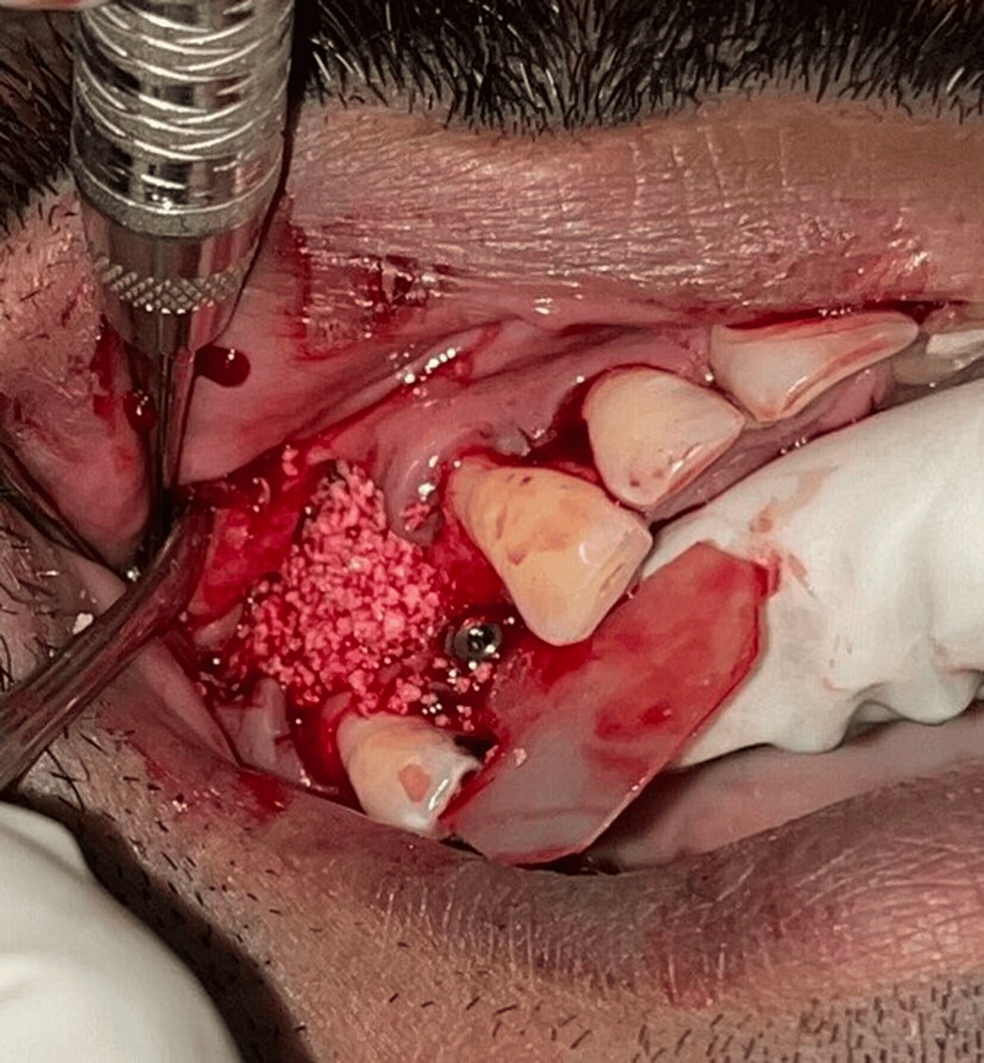
Case 1 - Bone graft and membrane placed on the buccal aspect of the ridge for horizontal ridge augmentation

A 46-year-old systemically healthy male was reported to the Department of Periodontology for a routine periodontal check-up. Upon clinical examination, an edentulous span was noticed in the region of 14, which had been extracted due to severe decay seven years ago. The patient demanded a dental implant-supported prosthesis. A ridge defect of H.1.i. according to Cologne classification of ridge defects [7] was identified (Figure 1). Soft tissue evaluation revealed a thin periodontal phenotype, shallow vestibule and inadequate KT in 15 regions. The patient was verbally explained about the comprehensive treatment plan. Hematologic parameters (CBC, PT, aPTT, BSL) were found to be within normal limits. A written informed consent was obtained. After administering local anaesthesia (Lignocaine with adrenaline; 1:2,00,000; LOX 2%) at the site an implant (B & B Dental Implant Company, Italy) of 4.5 x 10mm was placed (Figure 2). The buccal ridge defect (H.1.i) at the implant site was grafted with xenograft (Osseograft, Advanced Biotech Products Pvt Ltd., India) and resorbable collagen membrane (Healiguide, Advanced Biotech Products Pvt Ltd., India) for buccal contour augmentation (Figure 3). Passive primary closure at the grafted site was achieved by flap advancement using the periosteal release of the buccal flap and approximation using 4-0 non-resorbable sutures (Prolene, Ethicon India). Suture removal was done after two weeks. The wound healing was uneventful.

**Figure 4:**
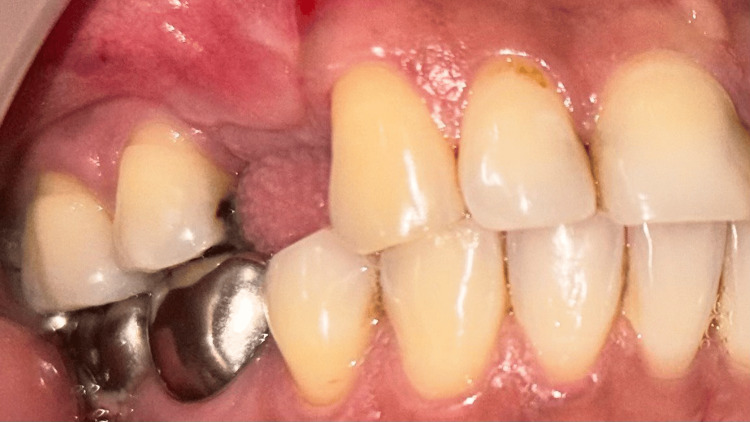
Case 1 - Insufficient keratinized tissue at the implant site after four months

**Figure 5:**
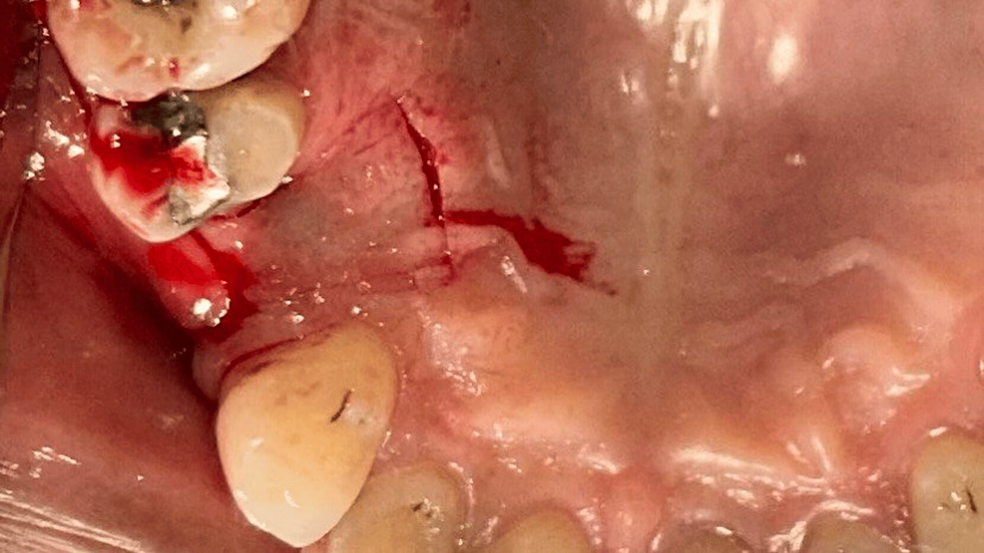
Case 1 - Horizontal incision given on palatal aspect and vertical papilla sparing incisions made from the edge of the palatal incision towards the buccal aspect

**Figure 6:**
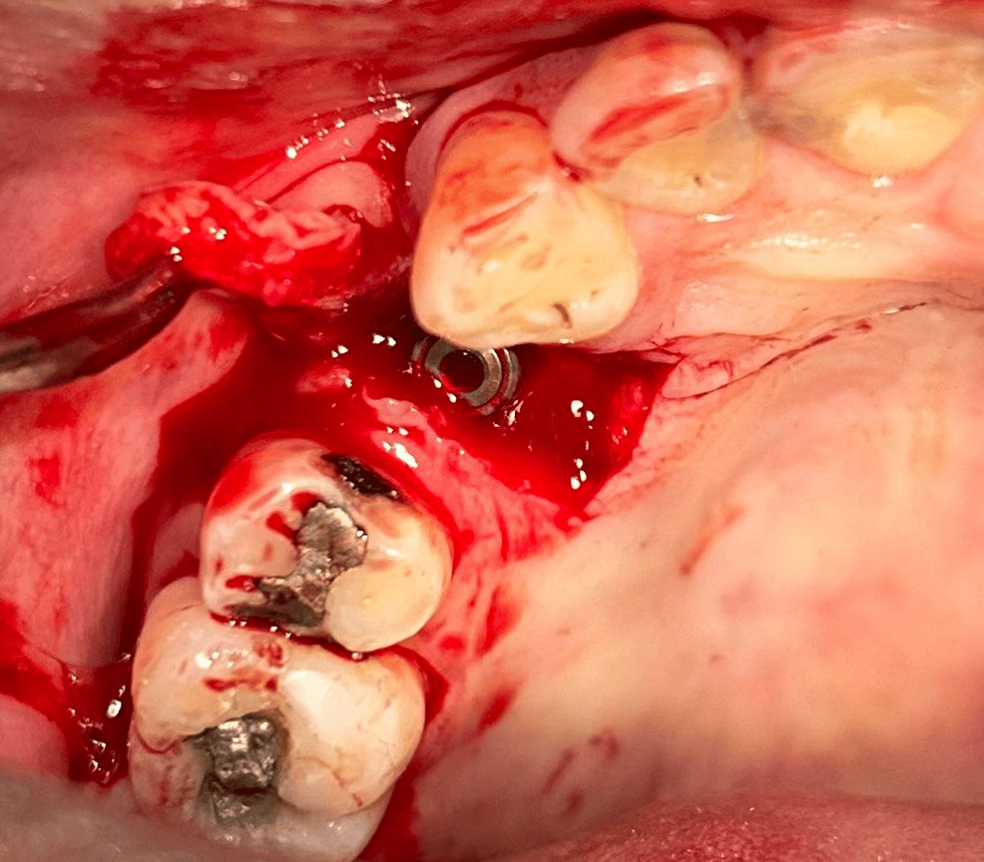
Case 1 - Flap reflected from palate to the buccal aspect

**Figure 7:**
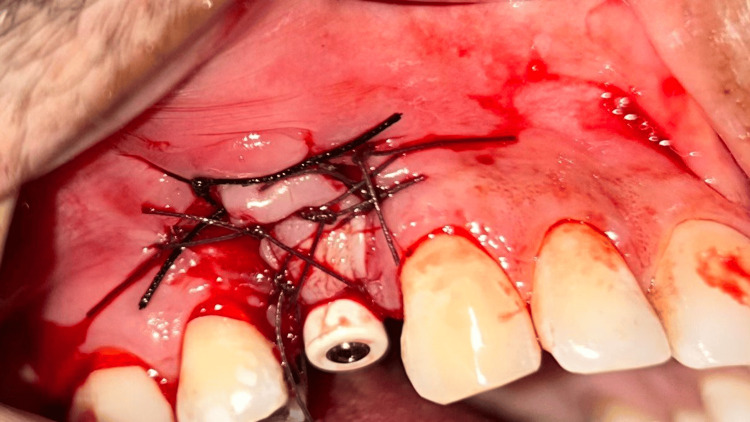
Case 1 - Buccal pedicled sliding flap sutured around healing abutment of adequate height

**Figure 8:**
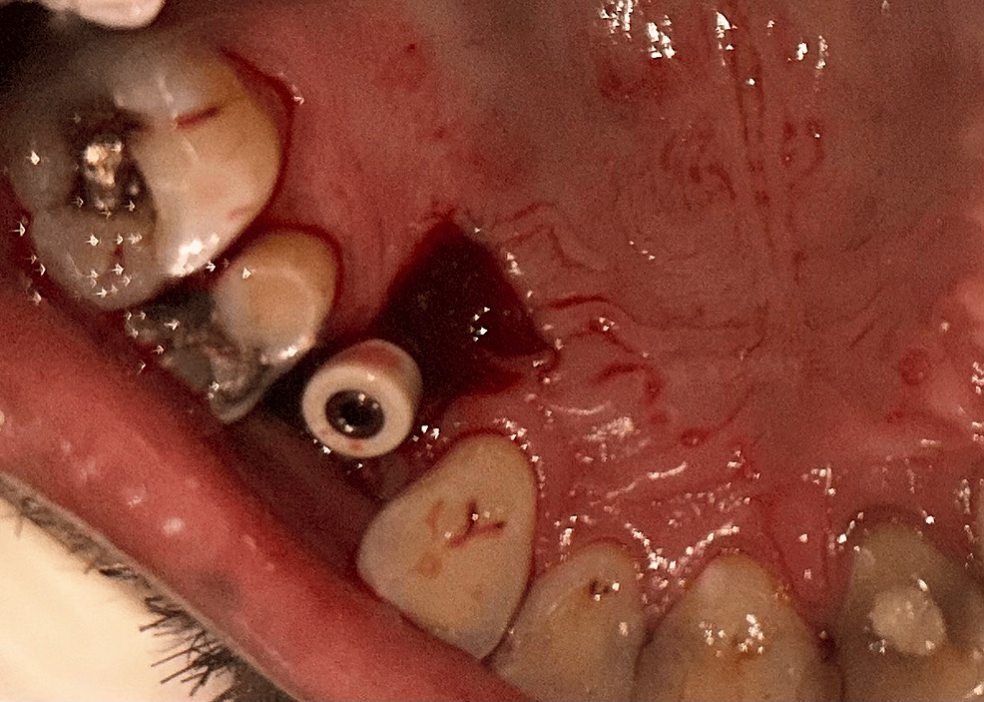
Case 1 - Palatal wound post-surgery

The patient was recalled after four months for second-stage surgery. Due to flap advancement for buccal contour augmentation, the width of KT at the implant site was found to be further reduced (Figure 4). Therefore, a minimally invasive technique using a buccal pedicled sliding flap was planned so as to reposition the palatal KT on the buccal aspect of the implant. After administering local anaesthesia (Lignocaine with adrenaline; 1:2,00,000; LOX 2%) at the site, a palatal incision was made approximately 8 mm from the mid-crestal line. Two vertical papilla-sparing incisions were made from the edges of the palatal incision (Figure 5) diverging and extending beyond the mucogingival junction on the buccal aspect. A partial thickness flap incorporating the palatal KT was raised from the palatal aspect to the mid-crestal area and extended as a full-thickness flap on the buccal aspect of the implant (Figure 6). A healing abutment of adequate height was placed on the implant. Flap was repositioned buccally transposing the KT onto the buccal aspect of the healing abutment (Figure 7). 5-0 resorbable sutures (Vicryl, Ethicon India) were placed to stabilize the flap. The palatal wound was allowed to heal by secondary intention (Figure 8).

Post-surgical Care

**Figure 9:**
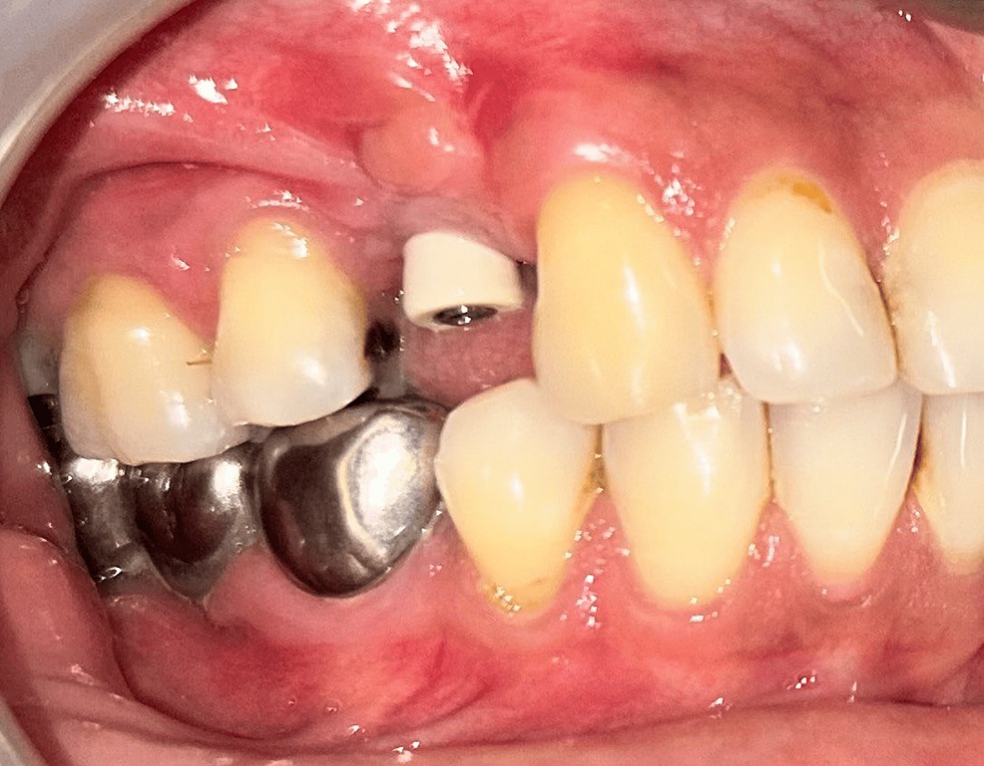
Case 1 - Adequate keratinized tissue acquired after one month (buccal view)

**Figure 10:**
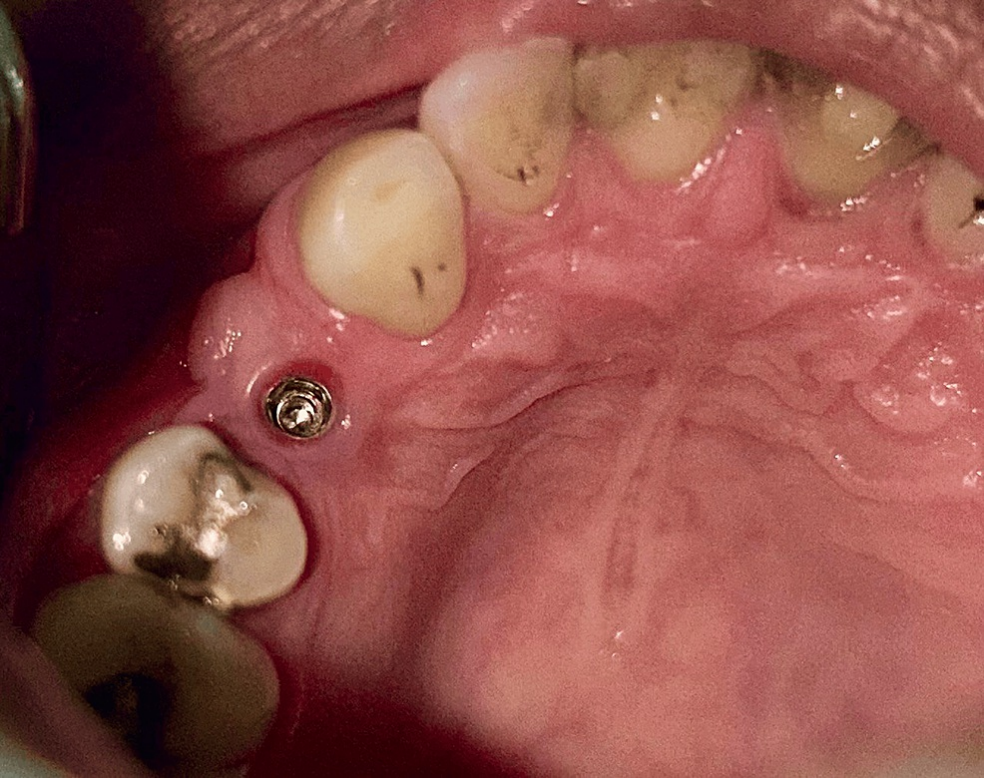
Case 1 - Soft tissue profile after one month (occlusal view)

**Figure 11:**
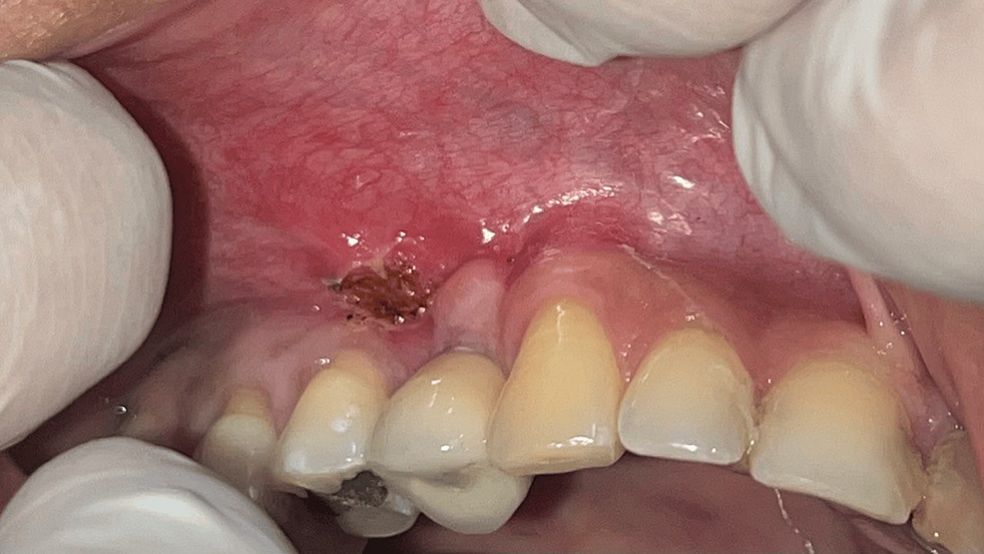
Case 1 - Buccal frenectomy done with diode laser

**Figure 12:**
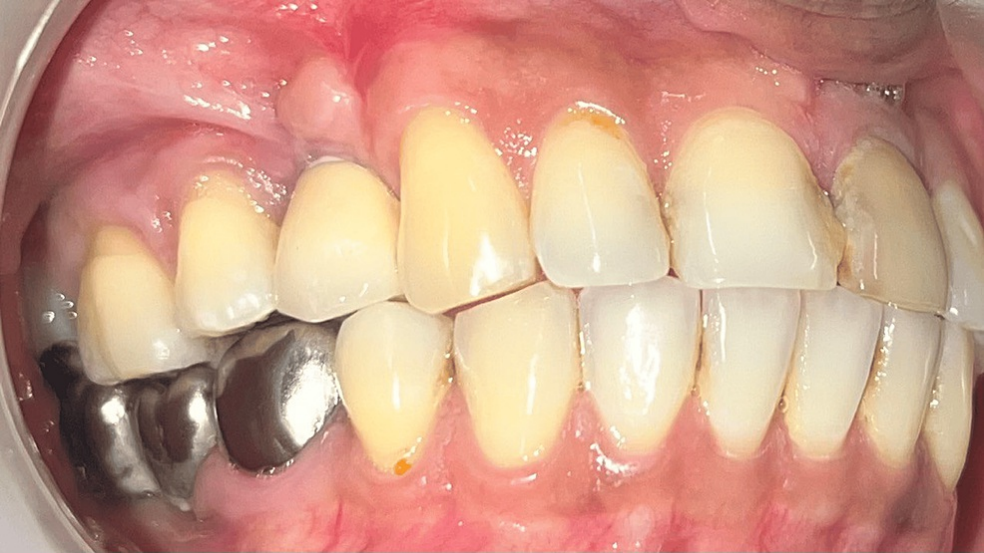
Case 1 - Porcelain fused metal (PFM) crown placed on the implant - 14

Post-operative instructions were given. Antibiotics (amoxicillin 625 mg), analgesics (diclomol, diclofenac sodium and paracetamol tablets) and anta-acid (pantoprazole 40 mg) were prescribed to the patient. Strict oral hygiene maintenance was also advised. Healing was found to be uneventful at four weeks follow-up. A thick band of KT had developed on the buccal aspect of the implant in 15 regions (Figures 9, 10). A frenal pull was noticed in the region of 15 which could affect the newly formed KT. Buccal frenectomy (Figure 11) was done using a Diode laser (Novolase Gold, 810 nm 2W; Gated pulsed mode, India). Prosthetic rehabilitation using porcelain fused metal crown was carried out (Figure 12).

Case 2

**Figure 13:**
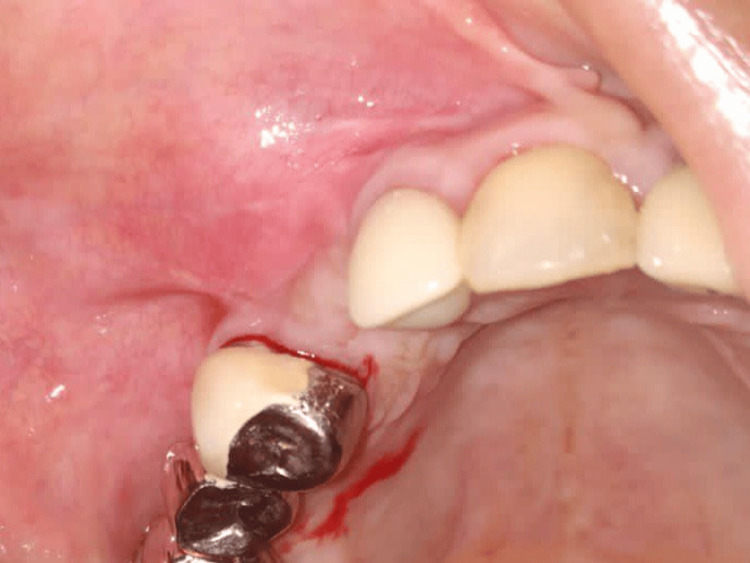
Case 2 - Inadequate keratinized tissue in 13 regions

**Figure 14:**
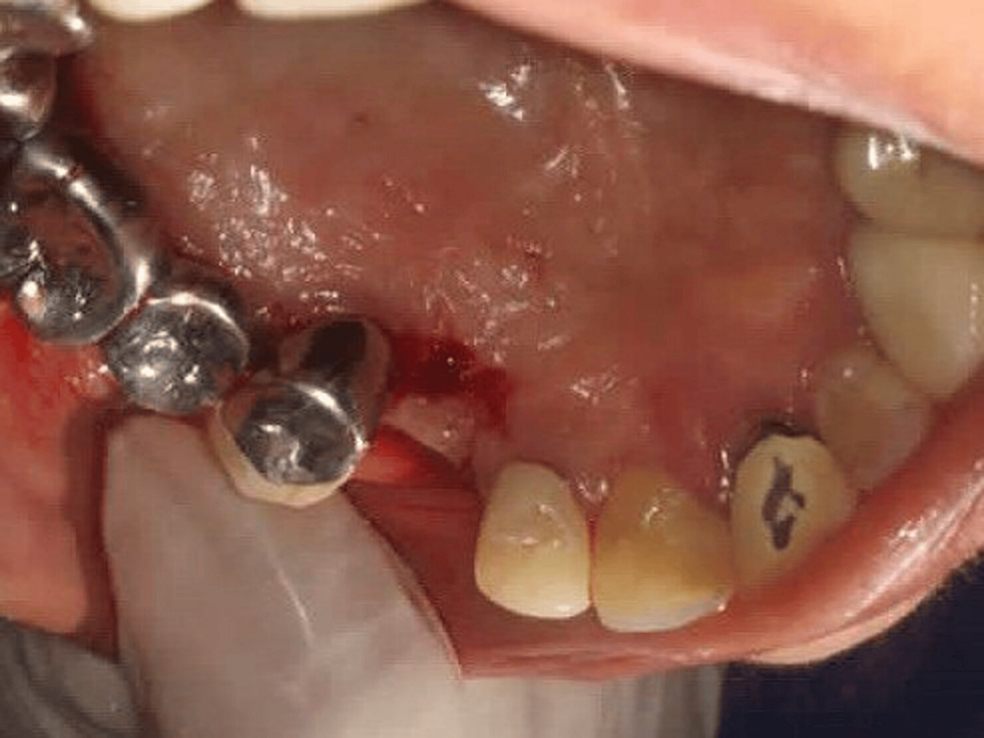
Case 2 - Horizontal incision given on the palate

**Figure 15:**
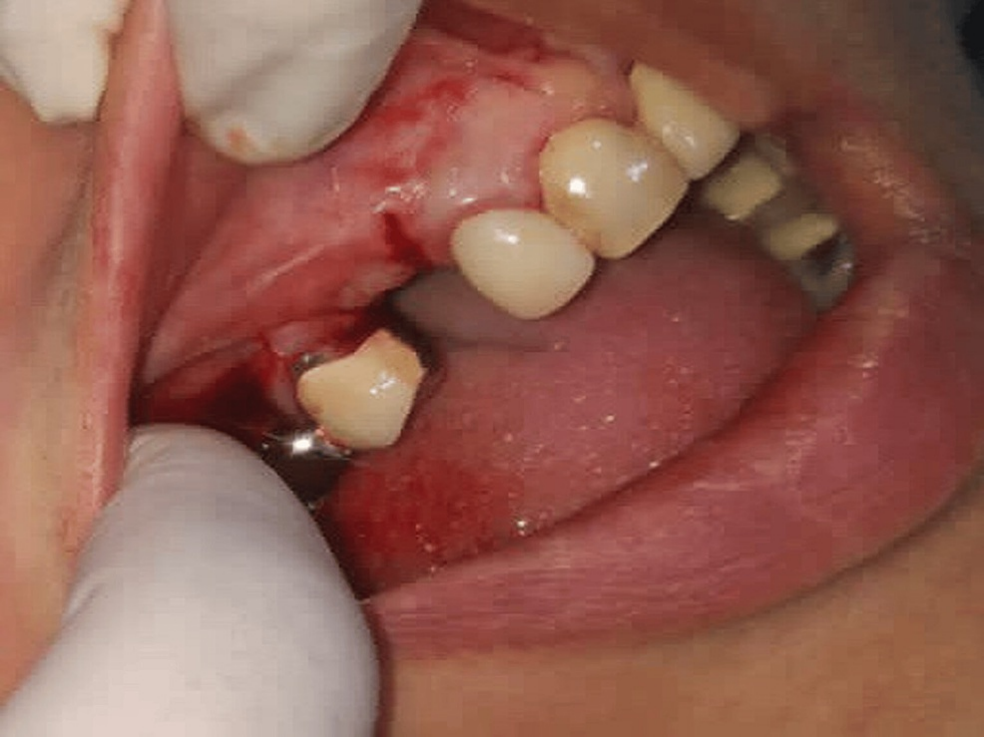
Case 2 - Papilla sparing incision given in 13 regions

**Figure 16:**
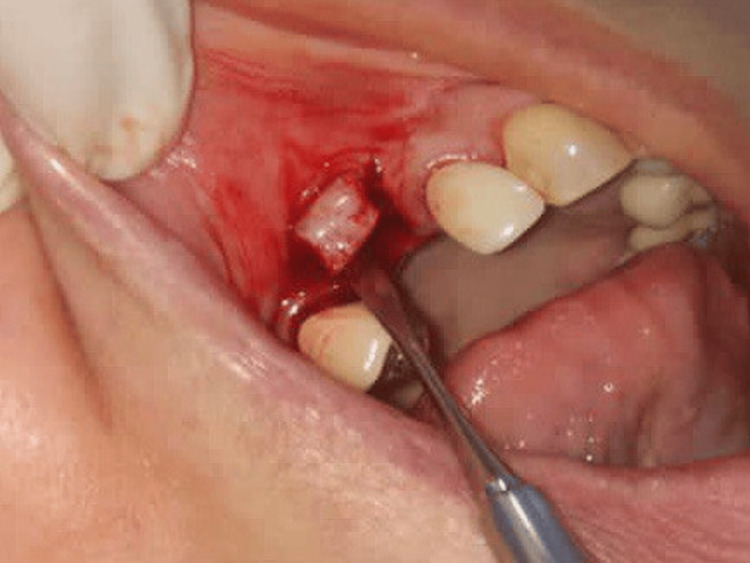
Case 2 - Flap reflected from palate on the buccal aspect

**Figure 17:**
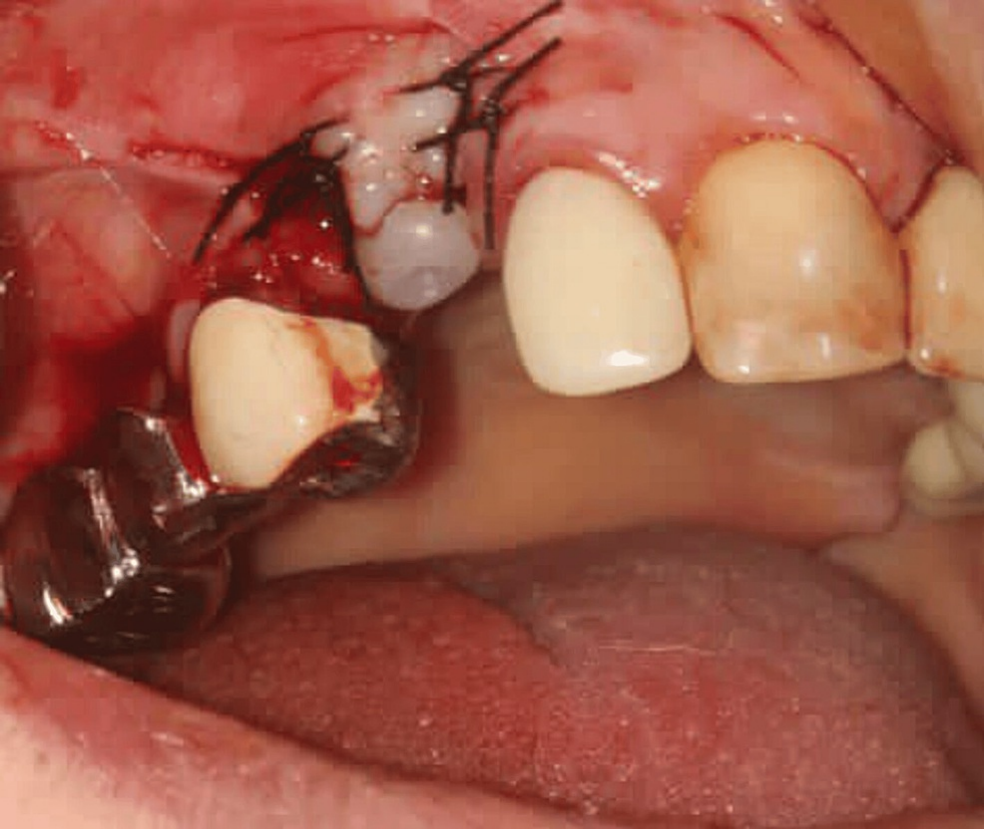
Case 2 - Buccal pedicle sliding flap sutured on the buccal aspect after abutment placement on the implant

**Figure 18:**
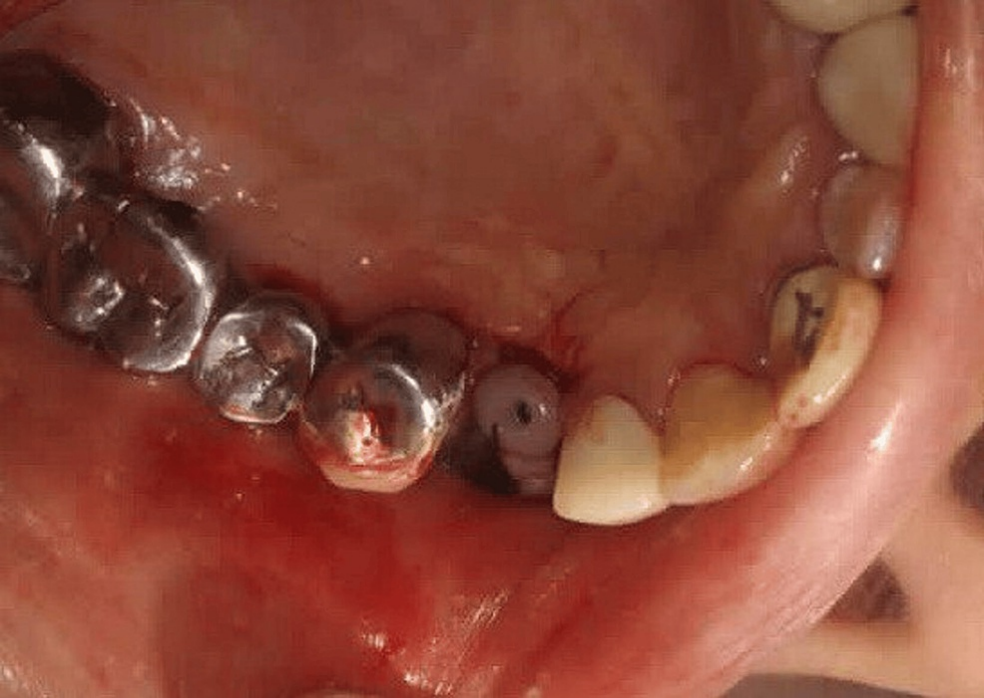
Case 2 - Palatal wound post-surgery

**Figure 19:**
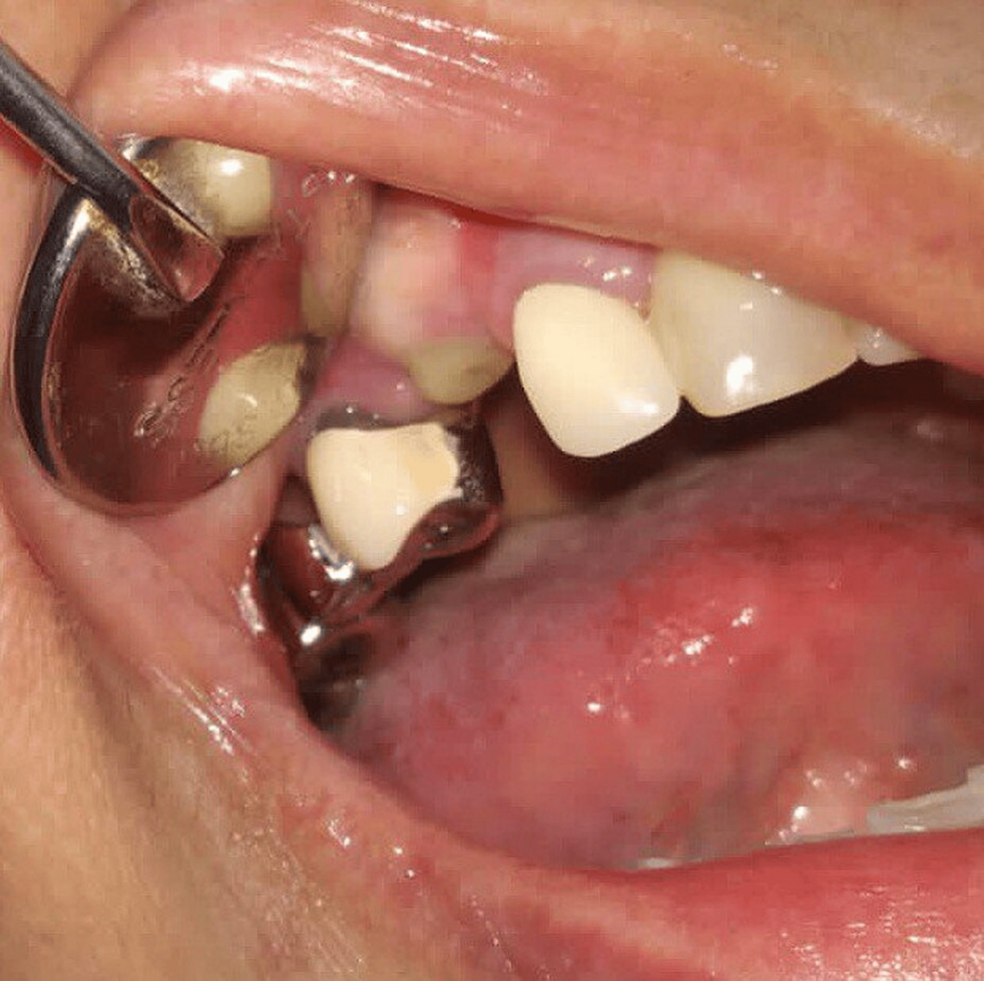
Post-op healing with adequate keratinized tissue

A 53-year-old, systemically healthy female patient was referred to the Department of Periodontology for soft tissue augmentation around an implant placed in 13 regions. Implant placement with buccal contour augmentation had been done four months back. Upon clinical examination thin, non-KT was found on the buccal aspect of the implant (Figure 13). The patient was verbally explained about the comprehensive treatment plan. Hematological investigations were found to be within normal limits. A written informed consent for soft tissue grafting was taken. A procedure with similar surgical steps as described in the above case was performed for KT augmentation during second-stage surgery around the implant in 13 regions (Figures 14-19). Post-surgical care was the same as mentioned in Case 1.

## Discussion

In today’s era, dental implants are commonly being used to rehabilitate partial or complete edentulism, not only to restore the function but also to achieve a natural-looking form and esthetics. Osseointegration remains crucial for the long-term stability of dental implants and a healthy peri-implant tissue is vital to achieve an emergence profile and esthetic blend with the adjacent gingival architecture. Preservation of soft tissue around osseointegrated implants remains an arduous task for clinicians. Peri-implant mucogingival surgery focuses on creating an emergence profile, improving the peri-implant tissue thickness [8,9], and achieving an adequate papillary height for excellent esthetic blending with the surrounding tissues.

Stable crestal bone levels are crucial for the longevity of dental implants [10]. Linkevicius T et al. (2015) in their prospective clinical trial concluded that thin mucosal tissues may cause early crestal bone loss, but their thickening with allogenic membrane may significantly reduce bone resorption. They further stated Implants in naturally thick soft tissues experienced minor bone remodelling [11].

A number of surgical techniques have been described to increase the amount of KT around dental implants. Soft tissue augmentation procedures can be performed at various time points, depending on the location of the implant and the complexity of the situation. The time points to achieve the most predictable outcomes are before implant placement and during (or after) the phase of osseointegration of the implant. The predominant techniques described are connective tissue graft (CTG), free gingival graft (FGG), and apically displaced flap [12,13]. Reddy et al. [14] suggested that an apically positioned flap (APF) yielded a significant improvement in KT (3.95 mm), which is both functionally and esthetically acceptable. Askin et al. [9] suggested in their clinical and radiographic longitudinal study that FGG performed around dental implants lacking KT is a reliable method. However, where esthetic outcome is the main priority, the systematic review by Thomas et al. concluded CTG is the best-documented method for gain of soft tissue volume at implant sites as it yields better papilla fill and higher marginal mucosal levels to non-grafted sites. FGG has certain disadvantages like donor site morbidity, unpredictable vascularization, etc. Pedicle grafts have the advantage of adequate blood supply which may accelerate tissue healing, reduce graft shrinking, and increase the chances of graft acceptance. Allogenic and xenogenic soft tissue grafts have also been used as other options for increasing peri-implant KT [15].

Techniques like the palatal roll flap technique [16] modified roll flap technique [17], rotated split palatal flap [18], and rotated double pedicle flap [19] have been successful in augmenting KTW around peri-implant tissues. However, these procedures are highly technique-sensitive, time-consuming and require specialized training.

Although minimally invasive techniques like flapless second-stage surgery i.e. key hole access expansion [20] or soft tissue punching, etc. are operator friendly these are futile when a thin layer of bone forms over the cover screw and needs to be removed to help place the healing abutment.

Our present article introduces a novel technique i.e. buccal pedicle sliding technique for boosting and volumising the soft tissue around dental implants during second-stage surgery. The proposed technique is easy, less technique-sensitive and takes relatively less time. Buccal sliding pedicle flap seems to be promising in terms of decreased morbidity, maintenance of the blood supply, stabilization of the pedicle, and superior homeostasis. Even if a thin layer of bone forms over the cover screw, this technique can provide access for bone removal to facilitate the placement of the healing abutment during second-stage surgery. By making use of abundant KT present on the palate, the desired outcome of adequate gain in KT around dental implants can be achieved using this minimally invasive surgical approach. This novel technique can be performed as one-stage or two-stage surgery and can be applied in anterior and posterior areas as well as at single and multiple adjacent implants. However, the limitation of this technique may be the open palatal wound which heals by secondary intention with no complications.

## Conclusions

Augmentation of peri-implant soft tissues is one the critical aspects for gaining the biological, functional and esthetic needs of the patient. Second-stage surgery should be given more emphasis rather than just placement of healing abutment. Modification of the periodontal phenotype can be done during this stage which may contribute to the proper contour and thickness while giving finesse to the peri-implant tissues. Further long-term studies to quantify the gain in KTW and KT thickness using this documented technique are warranted.
